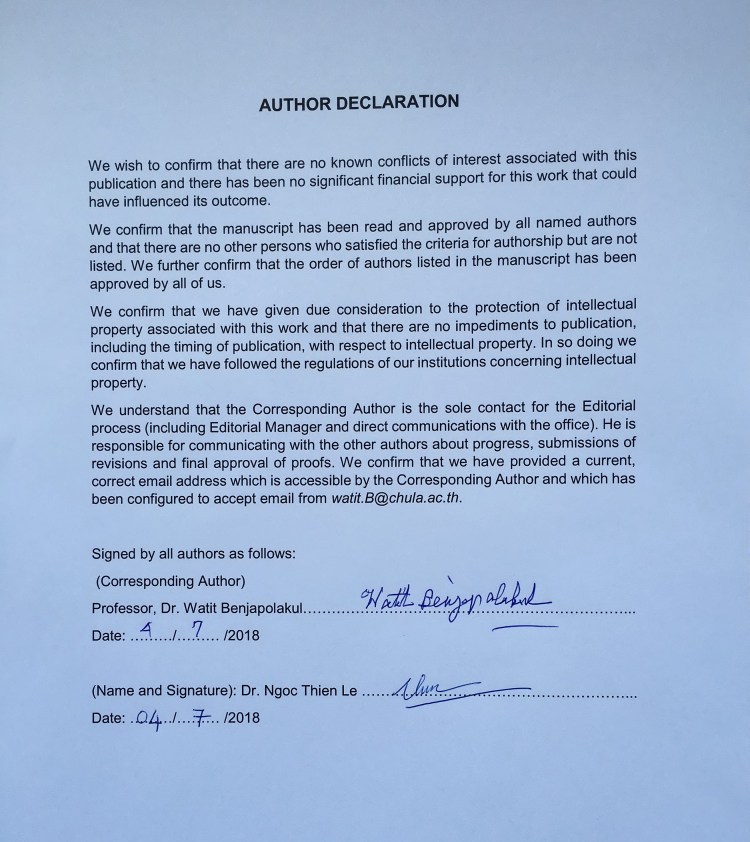# Received signal strength data of ZigBee technology for on-street environment at 2.4 GHz band and the interruption of vehicle to link quality

**DOI:** 10.1016/j.dib.2019.01.027

**Published:** 2019-01-18

**Authors:** Ngoc Thien Le, Watit Benjapolakul

**Affiliations:** Department of Electrical Engineering, Faculty of Engineering, Chulalongkorn University, Bangkok, Thailand

## Abstract

ZigBee technique is the common wireless network approaches for many smart indoor management applications such as home energy management or building management system. However, the application of ZigBee for outdoor applications is still not mature since the characteristic of signal strength depends on the outdoor conditions. To solve this issue, the received signal strength index (RSSI) and packet delivery are measured in real outdoor conditions for wireless neighborhood area network (WNAN) planning and optimization. Unfortunately, these important data are usually not publicly available for academic use. In this data article, RSSI data and packet delivery rate of a wireless network based on ZigBee 2.4 GHz are collected at the on-street condition, considering the effect of moving and stopping vehicles. In addition, the transmission ranges at two module location levels are also collected. The data provided in this article will help to estimate the transmission range, avoid the interferences and adjust the power level of ZigBee devices for many outdoor applications.

## Specifications table

**Table:** 

Subject area	*Electrical Engineering*
More specific subject area	*Wireless short-range communication*
Type of data	*Tables, graphs, and figures*
How data was acquired	*The loopback test measurements between a ZigBee master and a ZigBee slaver was conducted and the received signal strength (RSSI in dB* *m) measurements were collected at 2.4* GHz band. Both ZigBee devices are used the XBEE Pro-S2C, the commercial module of Digi International Inc. The modules are placed on the walking lane of a two-way street, at 1.2 m high from the ground.
Data format	*Raw, analyzed*
Experimental factors	*The loopback test mimicked the communication between a local controller station and a street lighting of a smart street lighting pilot project based on ZigBee at the same street. The parameters were adhered to as the suggested by this pilot project.*
Experimental features	*The statistical analyses and the probability distributions of the RSSI data are shown.*
	*The graphs are presented to show the interruption of moving and stopping vehicles to the link quality.*
	*A correlation analysis of the communication range and the time of interruption was performed to understand the relationship between to datasets.*
Data source location	*The data in this article were gathered along a two-way street Rat Burana Road (three lanes each side), Bangkok, Thailand (Latitude 6.47385*^*o*^*N, Longitude 3.00408*^*o*^*E).*
Data accessibility	*Datasets on received signal strength at many range tests are provided in this article in easily reusable format.*
Related research article	*Mujahid Tabassum and Kartinah Zen, Performance evaluation of ZigBee in indoor and outdoor environment. In: 9th International Conference on IT in Asia (CITA), 4–5 August, 2015, pp. 1–7.*

## Value of the data

• These data are needed by network engineers to understand the characteristics of ZigBee network at 2.4 GHz band used for many wireless outdoor applications such as smart street lighting network, traffic lighting network, smart electric meters network;
• This data provides valuable evidence about the impacts of running and stopping vehicles to the link quality of ZigBee network at 2.4 GHz band. Therefore, it is useful to consider for further tests and simulations;
• This dataset is a necessary input for the design and development of ZigBee signal propagation model, considering the interruption of vehicles.

## 1 Data

**Fig. 1:**
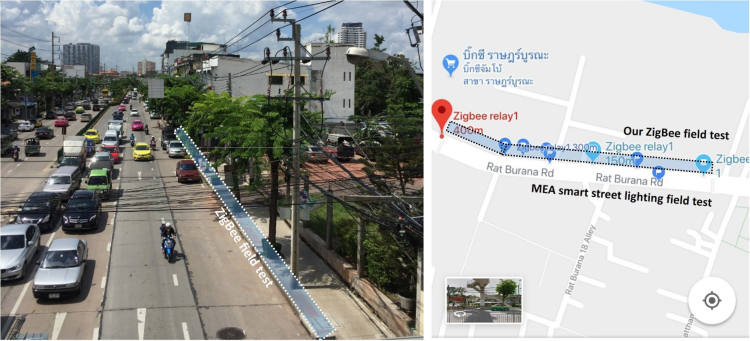
The test route, which includes a smart lighting test site is located on one walking lane of the street. All the sensors are located at one side of the street (blue area in picture).

**Fig. 2:**
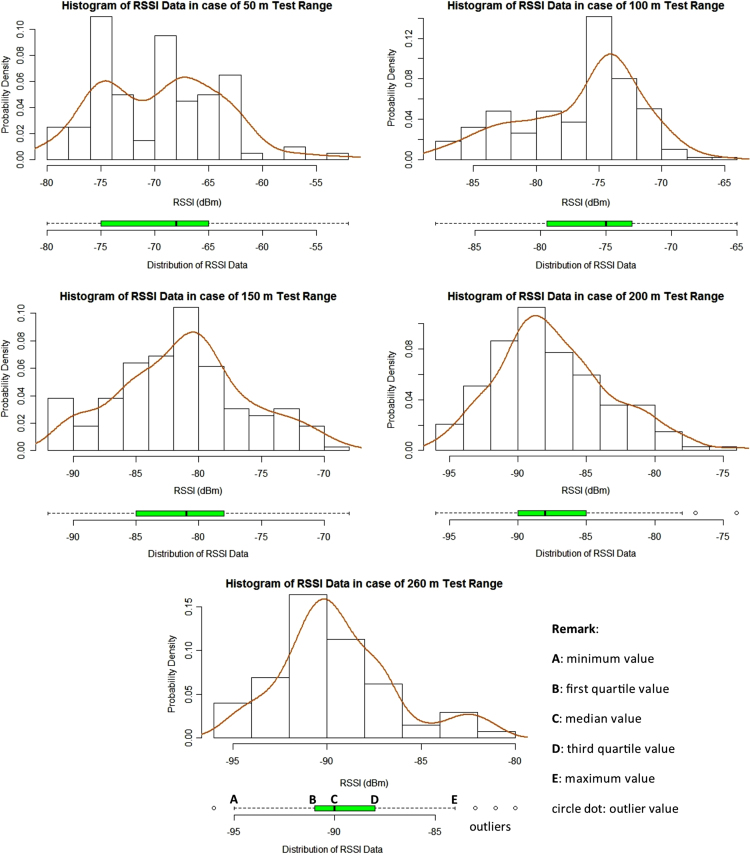
The histograms of RSSI data from loopback range tests in the same module level. The boxplot bar on bottom displays the distribution of RSSI data. The smooth curve uses Gaussian distribution as kernel density estimator.

**Fig. 3:**
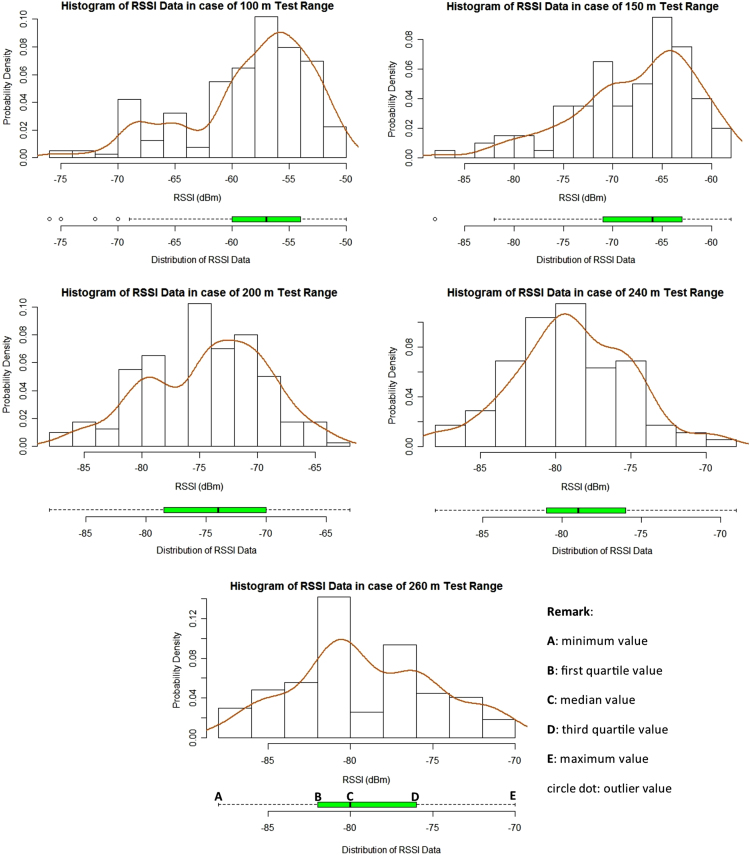
The histograms of RSSI data from loopback range tests in the different module level. The boxplot bar on bottom displays the distribution of RSSI data. The smooth curve uses Gaussian distribution as kernel density estimator.

**Fig. 4:**
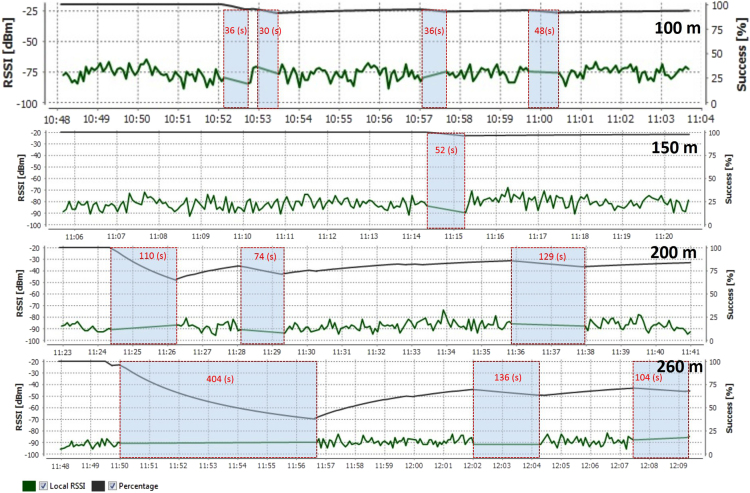
The interferences of stopping vehicles happened in range tests, same module level 1.2 m from the street ground.

**Fig. 5:**
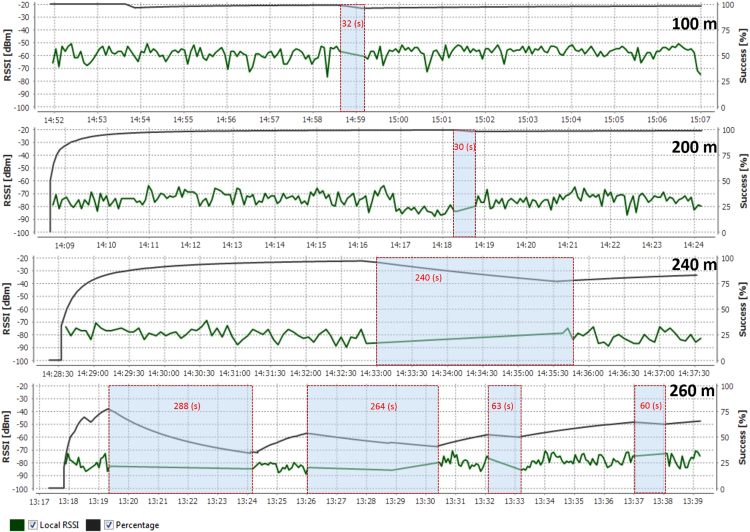
The interferences of stopping vehicles happened in range tests, different module level from the street ground.

In this data article, the received signal strength index (RSSI) data and the packet delivery ratio of loopback range test of ZigBee 2.4 GHz were measured along the Rat Burana road, Bangkok, Thailand (Fig. 1). The collected data included two scenarios. In the first one, both ZigBee transmitter and receiver were located at the same street level (1.2 m from the ground). In the second one, the transmitter was located at the pedestrian overhead bridge (4.5 from the ground) while the receiver node was still at 1.2 m. The descriptive statistics of RSSI data are represented in Table 1. Figs. 2 and 3 show histograms of received signal strength (RSSI) in dBm in the same module level and difference module level scenarios. The red rectangles Figs. 4 and 5 represent the interferences of stopping vehicles to the wireless link during the range tests.

**Table 1:** The descriptive statistics of RSSI data in the loopback test.

	Same level					Different level				
	**50 m**	**100 m**	**150 m**	**200 m**	**260 m**	**100 m**	**150 m**	**200 m**	**240 m**	**260 m**
Mean	− 69.4	− 76.4	− 81.1	− 87.3	− 89.4	− 58.3	− 67.7	− 74.3	− 78.9	− 79
Median	− 68	− 75	− 81	− 88	− 90	− 57	− 66	− 74	− 79	− 80
Mode	− 75	− 75	− 80	− 90	− 90	− 56	− 70	− 75	− 80	− 80
Standard Deviation	5.69	4.83	5.07	4.11	3.09	5.42	6.09	5.21	3.88	4.31
Variance	32.4	23.4	25.7	16.9	9.56	29.4	37.1	27.1	15	18.6
Kurtosis	2.74	2.64	2.66	3.05	3.44	3.23	3.37	2.5	3.13	2.38
Skewness	0.277	− 0.055	0.13	0.49	0.62	− 0.91	− 0.82	− 0.31	− 0.04	0.14
Range	28	23	24	22	15	26	30	25	19	18
Minimum	− 80	− 88	− 92	− 96	− 96	− 76	− 88	− 88	− 88	− 88
Maximum	− 52	− 65	− 68	− 74	− 81	− 50	− 58	− 63	− 69	− 70

## 2 Experimental design, materials and methods

### 2.1 Loopback range test

This test helps to evaluate the existing street lighting network based on ZigBee 2.4 GHz. This project is hosted by Metropolitan Electricity Authority (MEA) company, Bangkok, Thailand (Fig. 1). Since the communication between ZigBee modules takes place over the street condition, the quality of the wireless signal can be affected by many factors such as reflection of waves, interferences, line-of-sight issues, device׳s location.

The loopback test is a method used to perform the transmission of the links. This test involves sending data packets from the local ZigBee module to the remote one and waiting for the echo to be sent from the remote to the local.

The data collection and real-time analysis were performed with the use of the XCTU tool [1]. This tool counted the number of packets sent and received by the local module and measured the received signal strength value (RSSI). XCTU tool ran on a laptop Intel Core i3-2348M@2.30 GHz speed with 2GB RAM and Windows 7 operating system. The local module was connected to this laptop through USB cable. The remote module was power by a 10,000 Ah portable battery.

### 2.2 Parameter settings and scenarios

**Fig. 6:**
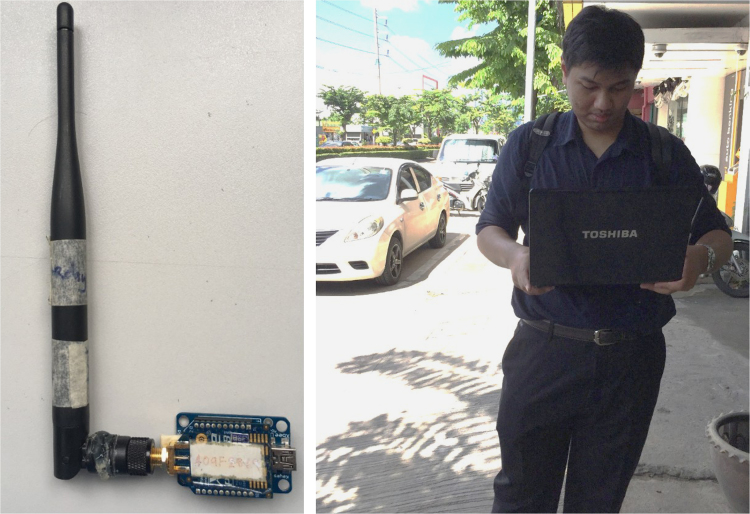
The Digi ZigBee Pro-S2C [2] used in our test (left); and the level 1.2 m of device during the test (right).
  ■ *Different level*: The remote module is located at the pedestrian overhead bridge with 4.5 m height from the street ground. The local module is at 1.2 m from the street ground.

Our field test is to support the MEA company to evaluate their smart street lighting pilot project at Rat Burana road. The field test route includes a traffic light in order to investigate the impact of running and stopping vehicles to the wireless link. The following parameters were adhered to as the suggested by this MEA:

• The *Rx_timeout = 10 (ms)*
  Local module timeout when sending packet to remote module. After the packet has been sent, the radio of local module switches into receiving mode and waits for 10 seconds for the remote to respond.
• The *Tx_interval = 400 (ms)*
  This is the maximum interval, in microseconds, that the local module would like to use when transmitting packets.

• Range test scenarios: There are two scenarios of the loopback range test.
  ■ *Same level*: Both local and remote modules are at 1.2 m height from the street ground (Fig. 6, right);

• An interference from stopping vehicles is identified when vehicles stop at the traffic light and the packet loss occurs in this period simultaneously.

### 2.3 Correlation coefficients between transmission range and interference

Table 2 presents the summary of number of interferences caused by stopping vehicles at traffic light. Table 3 shows the Pearson correlation coefficients and *P*-values to evaluate the relationship between transmission range and number of interferences in the statistic aspect. The transmission length and number of interferences correlate poorly, however, within our project, we intend to represent the general relationship of data we have collected. And further research study will be done to investigate this correlation.

**Table 2:** The summary of occurred interferences from stopping vehicles during the range tests.

**Scenario**	**transmission length (m)**	**Number of interferences**
Same module level (1.2 m from the street ground)	50	0
	100	4
	150	1
	200	3
	260	3
Different module level (Local module is 1.2 m from the street ground; remote module is 7.5 m from the street ground.)	100	1
	150	0
	200	1
	240	1
	260	4

**Table 3:** The Pearson correlation coefficients and P-values.

**Relationship**	**Correlation value (95% Confidence Interval)**	***P*-Value**
Transmission length (same level) and number of interferences	0.48 (CI.: − 0.70 to 0.95)	0.41
Transmission length (different level) number of and interferences	0.63 (CI.: − 0.57 to 0.97)	0.25

## Figures and Tables

**Fig. S1:**